# Effects of inosine on reperfusion injury after cardiopulmonary bypass

**DOI:** 10.1186/1749-8090-5-106

**Published:** 2010-11-08

**Authors:** Gábor Veres, Tamás Radovits, Leila Seres, Ferenc Horkay, Matthias Karck, Gábor Szabó

**Affiliations:** 1 Department of Cardiac Surgery, University of Heidelberg, Heidelberg, Germany; 2 Department of Cardiac Surgery, Semmelweis University, Budapest, Hungary; 3 Heart Center, Semmelweis University, Budapest, Hungary; 4 Gottsegen National Institute of Cardiology, Budapest, Hungary

## Abstract

**Objective:**

Inosine, a break-down product of adenosine has been recently shown to exert inodilatory and anti-inflammatory properties. Furthermore inosine might be a key substrate of pharmacological post-conditioning. In the present pre-clinical study, we investigated the effects of inosine on cardiac function during reperfusion in an experimental model of cardioplegic arrest and extracorporal circulation.

**Methods:**

Twelve anesthetized dogs underwent hypothermic cardiopulmonary bypass. After 60 minutes of hypothermic cardiac arrest, reperfusion was started after application of either saline vehicle (control, n = 6), or inosine (100 mg/kg, n = 6). Left ventricular end-systolic pressure volume relationship (ESPVR) was measured by a combined pressure-volume-conductance catheter at baseline and after 60 minutes of reperfusion. Left anterior descendent coronary blood flow (CBF), endothelium-dependent vasodilatation to acetylcholine (ACh) and endothelium-independent vasodilatation to sodium nitroprusside (SNP) were also determined.

**Results:**

The administration of inosine led to a significantly better recovery (given as percent of baseline) of ESPVR 90 ± 9% vs. 46 ± 6%, p < 0.05. CBF and was also significantly higher in the inosine group (56 ± 8 vs. 23 ± 4, ml/min, p < 0.05). While the vasodilatatory response to SNP was similar in both groups, ACh resulted in a significantly higher increase in CBF (58 ± 6% vs. 25 ± 5%, p < 0.05) in the inosine group.

**Conclusions:**

Application of inosine improves myocardial and endothelial function after cardiopulmonary bypass with hypothermic cardiac arrest.

## Background

Ischemia-reperfusion injury is a well-known phenomenon after cardiac surgery. Independent of the technique of cardioplegia, temporary dysfunction of biventricular contractility can frequently be observed. Even if cardiac dysfunction is not clinically evident, a reduction of myocardial contractility may occur as described in a study in humans using pressure-volume relationships [1]. In addition, coronary endothelial dysfunction may further complicate the postoperative course.

Extra-corporal circulation is also known to induce a systemic inflammatory reaction with free radical release leading to secondary organ injury. During ischemia, cellular ATP is degraded into AMP, adenosine, inosine and hypoxanthine. Adenosine and its primary metabolite inosine are ubiquitous nucleosides that can be released from ischemic or reperfused tissue [2]. Adenosine and adenosine analogues have been shown to act as an endogenous cardio-protective agent against ischemia-reperfusion injury [3,4]. Until recently, inosine was generally considered an inactive metabolite. Nevertheless, some past and recent works suggested that inosine may exert inotropic, vasodilatory and anti-inflammatory effects [5-7]. It has recently been discovered, that inosine inhibit poly (ADP-ribose) polymerase (PARP) activation [8]. It is also demonstrated, that PARP activation occurs during the reperfusion but not during the ischaemia [9].

As the use of inosine for prevention of reperfusion injury in the context of cardiopulmonary bypass has not yet been investigated, the aim of the present study was to test the hypothesis that treatment with inosine during reperfusion improves myocardial, and endothelial function in a clinically relevant canine model of extracorporal circulation.

## Methods

### Animals

12 dogs (foxhounds) weighing 21 to 35 kg (24.4 ± 1.5 kg) were used in these experiments. All animals received human care in compliance with the "Principles of Laboratory Animal Care" formulated by the National Society for Medical Research and the "Guide for the Care and Use of Laboratory Animals" prepared by the Institute of Laboratory Animal Resources and published by the National Institutes of Health (NIH Publication No. 86-23, revised 1996). The experiments were approved by the Ethical Committee of the Land Baden-Württemberg for Animal Experimentation.

### Surgical preparation and general management

The dogs were premedicated with propionylpromazine and anesthetized with a bolus of pentobarbital (15 mg/kg initial bolus and then 0.5 mg/kg/h i.v.), paralyzed with pancuronium bromide (0.1 mg/kg as a bolus and then 0.2 mg/kg/h i.v.) and endotracheally intubated. The dogs were ventilated with a mixture of room air and O_2 _(FiO_2 _= 60%) at a frequency of 12-15/min and a tidal volume starting at 15 ml/kg per minute. The settings were adjusted by maintaining arterial partial carbondioxide pressure levels between 35-40 mmHg. The femoral artery and vein were cannulated for recording mean arterial pressure (MAP) and taking blood samples for the analysis of blood gases, electrolytes and pH, and parameters of blood coagulation. Basic intravenous volume substitution was carried out with Ringer's solution at a rate of 1 ml/min/kg. If necessary, the rate of volume substitution was modified according to the continuously controlled input-output balance in order to maintain cardiac output at baseline levels. According to the values of potassium, bicarbonate and base excess, substitution included administration of potassium chloride and sodium bicarbonate (8.4%). Neither catecholamines nor other hormonal or pressor substances were administered. Rectal temperature and standard peripheral electrocardiogram were monitored continuously.

After left anterolateral thoracotomy in the fourth intercostal space, pericardiotomy and isolation of the great vessels a perivascular ultrasonic flow probe was attached to the ascendent aorta. Aortic pressure was monitored with 5F Millar catheter tip manometer (Millar Instruments, Inc., Houston, Tex).

### Cardiopulmonary bypass

After systemic anticoagulation with sodium heparin (300 U/kg) the left subclavian artery was cannulated for arterial perfusion. The venous cannula was placed in the right atrium. The extracorporeal circuit consisted of a heat exchanger, a venous reservoir, a roller pump and a membrane oxygenator primed with Ringer lactate solution (1000 ml) supplemented with heparin (150 U/kg) and 20 mL sodium bicarbonate (8,4%). Hypothermic CPB was performed for 90 minutes at a lowest temperature of 28 °C. After initiation of CPB, the hearts were arrested with 1000 ml crystalloid cardioplegia (Custodiol). Twenty minutes before cross-clamp removal, rewarming was initiated. After 60 minutes the aortic crossclamp was released and the reperfusion phase was initiated. All animals were weaned from CPB without inotropic support. After weaning from CPB, heparin was antagonized by protamine iv over 10 minutes and the animals were monitored for one hour. Thereafter, the hearts were excised for further investigation.

Twenty minutes before cross-clamp removal, rewarming was initiated. After 60 min of cardiac arrest, the aorta was declamped, and the heart was reperfused with normothermic blood in the bypass circuit. If necessary, ventricular fibrillation was counteracted with DC cardioversion of 40 J. Ventilation was restarted with 100% oxygen. All animals were weaned from CPB without inotropic support 20 min after the release of the aortic cross clamp. Each animal underwent 90 min of CPB with 60 min of cardiac arrest.

### Experimental groups

Two groups of animals were studied. Group 1 (n = 6) received placebo, group 2 (n = 6) received 100 mg/kg inosine during reperfusion. The applied dose of inosine is based on previous dose-response and pharmacokinetic studies.

### Measurements

Left and right ventricular systolic pressure (LVSP, RVSP), maximum and minimum pressure development (+dP/dt, -dP/dt), end-diastolic pressure (LVEDP, RVEDP) and cardiac output (CO) as the equivalent of aortic flow were monitored continuously. Stroke volume (SV) was calculated from the integrated flow signal and was used to calibrate the volume signal from the conductance catheter. Parallel conductance was estimated by rapid injection of one ml of hypertonic saline into the pulmonary artery or vena cava superior, respectively.

The volume signal provided by the conductance catheter was registered continuously (Sigma F5, Leycom, Leiden, The Netherlands) and computed by the Conduct PC software (Leycom, Leiden, The Netherlands). Left and right ventricular pressure-volume loops were constructed on-line. Vena cava occlusions were performed to obtain a series of loops for calculation of the slope (Ees) and intercept (V0) of the left and right ventricular end-systolic pressure-volume relationships. In addition, the slope of the left ventricular end-systolic pressure-volume relationship (ESPVR) and preload recruitable stroke work (PRSW) were calculated as load-independent indices of myocardial contractility.

Coronary blood flow (CBF) was measured by an ultrasonic flow meter placed on the left anterior descendent coronary artery. Coronary endothelium-dependent vasodilatation was assessed after intracoronary administration of a single bolus of acetylcholine (ACh, 10^-7 ^mol) and endothelium-independent vasodilatation after administration of sodium-nitroprusside (SNP, 10^-4 ^mol). The vasoresponse was expressed as percent change of baseline coronary blood flow.

### Data analysis

All measurements were performed before cardiopulmonary bypass and after 60 min of reperfusion. All values were expressed as mean * standard error of the mean (SEM). Paired t-test was used to compare two means within groups. Individual means between the groups were compared by one-way analysis of variance followed by an unpaired t-test with Bonferroni correction for multiple comparisons and the post-hoc Scheffe's test. A probability value less than 0.05 was considered statistically significant. In the figures only the significances between the groups were indicated. Significant changes over the time within each group were indicated in the text.

## Results

### Hemodynamic parameters

Hemodynamic variables are shown in Table 1. Baseline parameters did not differ between the groups and were within the physiological range. HR did not change either in the control or in the inosine group. After 60 min of cardioplegic arrest and 60 min of reperfusion, MAP decreased significantly (*p *< 0.05) in the control group while it remained unchanged in the inosine group. CO did not differ significantly between the groups. However, it should also be noted that CO showed a clear decreasing tendency within the control group without reaching the level of significance [Table 1].

**Table 1:** Hemodynamic variables before cardiopulmonary bypass and after 60 minutes of reperfusion.

	Baseline		60 minutes of reperfusion	
	**control**	**inosine**	**Control**	**inosine**

HR [beats/min]	119 ± 6	108 ± 8	129 ± 9	122 ± 7

MAP [mmHg]	96 ± 8	89 ± 9	67 ± 6°	81 ± 6*

CO [l/min]	2.62 ± 0.32	2.34 ± 0.51	2.10 ± 0.42	2.32 ± 0.77

°p < 0.05 versus before CPB, *p < 0.05 versus control.

### Left and right ventricular function

**Figure 1:**
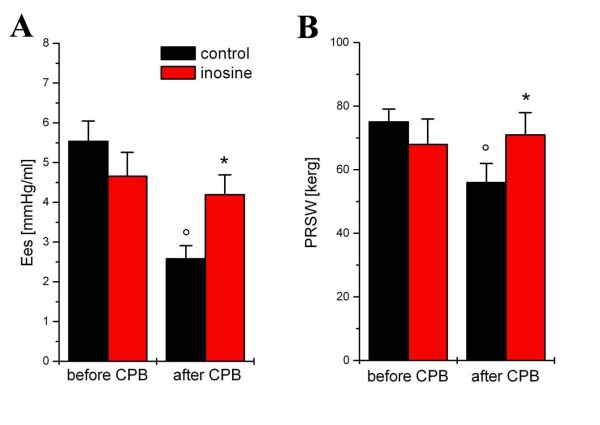
**The effect of inosine on left ventricular contractility**. The slope (Ees) of left ventricular end-systolic pressure-volume relationships (A) and preload recruitable stroke work (PRSW) (B) are shown in control and inosine-treated dogs. All values are given as mean ± SEM, *p < 0.05 vs. control, °p < 0.05 vs. before CPB.

**Figure 2:**
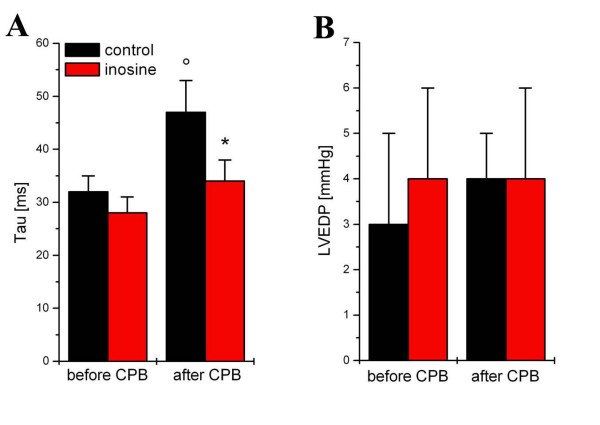
**The effect of inosine on diastolic cardiac function**. Time constant of lerft ventricle decay (Tau) (A) and left ventricle end-diastolic pressure (LVEDP) (B) are shown in control and inosine-treated dogs. All values are given as mean ± SEM, *p < 0.05 vs. control, °p < 0.05 vs. before CPB.

Left ventricular function was identical in both groups at baseline [Figure 1]. Myocardial contractility-characterized by the load-independent slopes Ees and PRSW [Figure 1] -showed a significant decrease (p < 0.05) after extracorporal circulation and reperfusion in the control group while it remained unchanged in the inosine-treated group. Representative pressure-volume loops are shown in Figure 1. Myocardial relaxation constant τ increased significantly (p < 0.05) in the control group at 60 min of reperfusion, but it remained at baseline level in the inosine group [Figure 2].

### Coronary blood flow and vascular function

**Figure 3:**
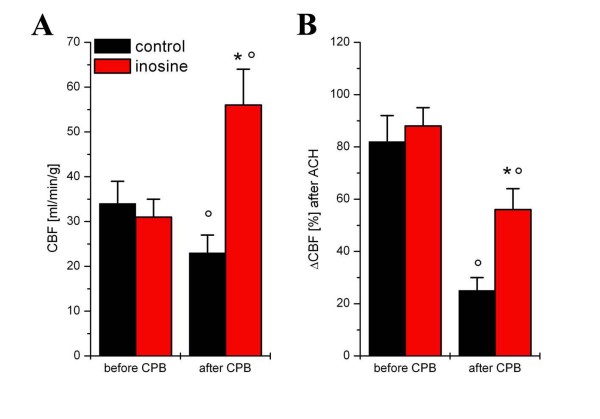
**Coronary endothelial function *in vivo***. Coronary blood flow (CBF) before and after 60 minutes of reperfusion (A). Endothelium-dependent vasodilatation after intracoronary administration of a single bolus of acetylcholine (10^-7 ^mol) expressed as percent change of coronary blood flow before and after cardiopulmonary bypass (CPB) at 60 min of reperfusion (B). All values are given as mean ± SEM; °p < 0.05 versus before CPB, *p < 0.05 versus control.

Coronary blood flow was similar in both groups before cardioplegic arrest. After 60 min of reperfusion, coronary blood flow decreased significantly (*p *< 0.05) in the control group, but it increased in the inosine group [Figure 3]. Endothelium-dependent vasodilatation after ACh was significantly (p < 0.05) reduced in the control group after 60 min of reperfusion in comparison to values before extracorporal circulation [Figure 3] but remained unchanged in the inosine group. Endothelium-independent vasodilatation after SNP showed no significant differences over time or between groups [data not shown].

## Discussion

In this study, the benefits of the application of inosine during reperfusion were assessed after cardiac arrest in a canine model of extracorporal circulation. We have shown that inosine improves left ventricle and endothelial function recovery after cardioplegic arrest.

Until recently, inosine was considered an inactive purine metabolite in most biological systems, but several recent studies have shown that it has immuno-modulatory [10], neuroprotective [11], cardioprotective [12] and overall cytoprotective effects. Furthermore, some other studies reported that extracellular inosine has powerful cellular protective effects. For example, inosine decreases the release of intracellular enzymes from hypoxic lymphocytes [13], improves renal function during ischemia [14] and inhibits inflammatory cytokine production [7]. Administration of inosine has also been shown to improve myocardial function during acute left ventricular failure [15,16] and improve myocardial and endothelial function after heart transplantation [12]. Despite the growing evidence of protective effects of inosine, the use of inosine for prevention of reperfusion injury in the context of cardiopulmonary bypass has not yet been investigated.

The most cardiac surgical procedures require cardiopulmonary bypass and cardioplegia, which causes ischemia-reperfusion injury. Ischemia-reperfusion injury initiates pathophysiological cascades including an inflammatory response with liberation of cytokines and free radicals. Triggered by peroxynitrite-induced DNA single-brand breaks, PARP catalyzes an energy-consuming polymerization of ADP-ribose, resulting in NAD depletion, inhibition of glycolysis, and the reduction of intracellular high-energy phosphates in the reperfused heart. In various types of ischemia-reperfusion, the prevention of PARP activation results in better preservation of the high-energy phosphates, resulting in an improved energy status [17-19]. It is also demonstrated, that PARP activation occurs during the reperfusion but not during the ischaemia [20]. Our group demonstrated previously, that inosine inhibits PARP activation in vivo and therefore modulates oxidant-induced cell death [12]. To the best of our knowledge, this was the first study that showed the effectiveness of inosine during the reperfusion in a clinically relevant large animal model of cardiopulmonary bypass. Administration of inosine during the reperfusion improved both systolic and diastolic indices of left ventricular contractility. In the current study, the load-independent indices such as preload recruitable stroke work (PRSW) and the slope of end-systolic pressure-volume relationship (ESPVR) of the left ventricle in the inosine group remained practically unchanged when compared with the baseline to 60-minutes reperfusion values. It is also well known, that the active phase of ventricle relaxation is an energy consuming phase of the cardiac circle, much like that of contraction. In the present study, we demonstrated that reperfusion injury is associated with impaired cardiac relaxation and diastolic dysfunction, as reflected by prolonged time constant of pressure decay (Tau) and increased LVDP. The decreased value of Tau in the inosine group clearly showed that inosine may significantly improve left ventricular diastolic function after CPB.

The increase of coronary blood flow during reperfusion contributes also to the improvement of cardiac function. Previous studies demonstrated that inosine increased coronary flow dose-dependently and, as a consequence, the function of the reperfused heart [21,22]. Our present data is in direct correspondence with these studies showing significant decrease of the coronary blood flow in the control group and unchanged CBF-values in the inosine group after reperfusion. This effect is comparable to those of with application of nitric oxide donors [23], endothelin receptor antagonists [24], or PARP-inhibitors during CPB [9,19,25]. How inosine protects the endothelium remains not completely understood. Previous data suggest that energy depletion severely impairs endothelial function [19]. It also demonstrated that adenosine and inosine breakdown may present an energy source to be preferred over extracellular glucose under hypoxia, as they delay the accumulation of NADH+ H^+^, thereby maintaining some vital cellular functions. Inosine may exert some of its cytoprotective effects under ischemia by providing an emergency energy source, when glucose is insufficient to support cellular functions. The above hypothesis is supported both by several reports that cellular ATP levels were elevated in ischaemic or hypoxic cells treated with adenosine or inosine [26-30] As inosine restores ATP levels, this may contribute to improved endothelial function. If inosine has a direct effect on nitric oxide synthesis remains to be clarified.

## Conclusions

In summary the results of this study showed that treatment with inosine during the reperfusion markedly improved post-ischemic myocardial and endothelial function after cardioplegic arrest in the setting of a cardiopulmonary bypass. Based on the promising data of the present study, inosine is supposed to utilize in the clinical usage. However, further clinical investigations are needed.

## Competing interests

The authors declare that they have no competing interests.

## Authors' contributions

All authors have made substantial contributions to conception and design, or acquisition of data, or analysis and interpretation of data; have been involved in drafting the manuscript or revisiting it critically for important intellectual content and have given final approval of the version to be published.
